# *Borreliae* Part 2: *Borrelia* Relapsing Fever Group and Unclassified *Borrelia*

**DOI:** 10.3390/biology10111117

**Published:** 2021-10-29

**Authors:** Giusto Trevisan, Marina Cinco, Sara Trevisini, Nicola di Meo, Maurizio Ruscio, Patrizia Forgione, Serena Bonin

**Affiliations:** 1 DSM—Department of Medical Sciences, University of Trieste, 34149 Trieste, Italy; trevisan@units.it (G.T.); ndimeo@units.it (N.d.M.); 2 DSV—Department of Life Sciences, University of Trieste, 34149 Trieste, Italy; marinacinco02@gmail.com; 3 ASUGI—Azienda Sanitaria Universitaria Giuliano Isontina, 34129 Trieste, Italy; sara.trevisini@asugi.sanita.fvg.it (S.T.); maurizio.ruscio@asugi.sanita.fvg.it (M.R.); 4 UOSD Dermatologia, Centro Rif. Regionale Malattia di Hansen e Lyme, P.O. dei Pellegrini, ASL Napoli 1 Centro, 80145 Naples, Italy; patriziaforgione2@gmail.com

**Keywords:** *Borrelia*, soft-tick-borne relapsing fever, hard-tick-borne relapsing fever, Old World strains, New World strains, louse-borne relapsing fever, avian relapsing fever, unclassified *Borreliae*, *Spirochaeta*

## Abstract

**Simple Summary:**

Following *Borreliae* Part 1, this second review describes *Borreliae* of the relapsing fever group (RFG) and unclassified *Borreliae*. The RFG is further divided according to vector transmission and geographical distribution in other subgroups, namely the soft-tick-borne relapsing fever (STBRF) group, the hard-tick-borne relapsing fever (HTBRF) one, the louse-borne relapsing fever (LBRF) group, and the Avian relapsing fever group. Where possible, according to the literature description, each sub-group of the RFG *Borreliae* is organized here in sections explaining the geographical distribution, the vectors, the hosts, the epidemiology, and the microbiology. In case of human infectiveness, clinical aspects are also discussed. Isolation and sequencing of *Borrelia* species is ongoing, and in addition to the groups mentioned in these reviews, there are *Borreliae* that at present cannot be cultivated, but according to sequencing data they share some characteristics with one specific group. Nevertheless, they still cannot be classified in one of them. This is the case of *Borreliae* with not-yet-identified pathogenicity for humans or animals, which are here named “unclassified *Borreliae*” and described separately, recalling the similarities with *Borreliae* already classified. In the future, we expect that those *Borreliae* are going to be characterized, including them in one of the previous groups.

**Abstract:**

*Borreliae* of the relapsing fever group (RFG) are heterogenous and can be divided mainly into three groups according to vectors, namely the soft-tick-borne relapsing fever (STBRF) *Borreliae*, the hard-tick-borne relapsing fever (HTBRF) *Borreliae*, the louse-borne relapsing fever (LBRF) *Borreliae*, and the avian relapsing fever ones. With respect to the geographical distribution, the STBRF *Borreliae* are further subdivided into Old World and New World strains. Except for the Avian relapsing fever group *Borreliae*, which cause avian spirochetosis, all the others share infectivity in humans. They are indeed the etiological agent of both endemic and epidemic forms of relapsing fever, causing high spirochaetemia and fever. Vectors are primarily soft ticks of *Ornithodoros* spp. in the STBRF group; hard ticks, notably *Ixodes* sp., *Amblyomma* sp., *Dermacentor* sp., and *Rhipicephalus* sp., in the HTBRF group; and the louse *pediculus humanus humanus* in the TBRF one. A recent hypothesis was supported for a common ancestor of RFG *Borreliae*, transmitted at the beginning by hard-body ticks. Accordingly, STBRF *Borreliae* switched to use soft-bodied ticks as a vector, which was followed by the use of lice by *Borrelia recurrentis*. There are also new candidate species of *Borreliae*, at present unclassified, which are also described in this review.

## 1. Introduction

*Borrelia* species are part of the Spirochaetaceae family; therefore, they are characterized by a spiral shape. Spirochaetes cause many important diseases in humans, including relapsing fever (RF), which is a widespread bacterial disease caused by microaerophilic spirochetes of the genus *Borrelia*. Most *Borrelia* spp. are transmitted by ticks (endemic forms), while the epidemic forms of relapsing fever are caused by body lice, through scratching that induces the rupture of the louse and micro skin wounds. *Borrelia* can be transmitted by soft ticks or by hard ticks. Depending on the geographic area and vectors, many *Borrelia* spp. can infect humans. Those types of Borreliosis are not clinically easy to distinguish from other febrile diseases, such as malaria in Africa [1].

The first description of “relapsing fever” was made by Hippocrates in 430 B.C. on the island of Thasos in the Northern Aegean Sea: “The vast majority (of sufferers) had a seizure on the sixth day, with an interval of six days followed by a seizure on the fifth day after relapse” [2]. Other typical features of the louse-borne relapsing fever (LBRF) were severe rigors, jaundice, profuse epistaxis, and a tendency to precipitate abortion [3]. Several episodes of “epidemic fever” in the following centuries were due to relapsing fever, based on the symptoms of the disease. The “yellow plague” that swept Europe in 550 (Justinian plague), of which the distinguishing feature was jaundice, was also probably predominantly RF transmitted by lice [4]. In Dublin in 1770, Rutty described a “fever lasting six or seven days, with multiple relapses” [5]. An epidemic of RF in 1812 struck one third of Napoleon’s Grande Armée in Vilnius, on the route from Moscow to Warsaw [6]. In Edinburgh, during the epidemic, which involved Ireland, Craigie in 1843 distinguished this infection transmitted by lice (*Pediculus humanus corporis*) from typhus and coined the name of “relapsing fever” [7]. This infection spread throughout the British Isles [8]. David Livingstone described fatal tick-borne RF in Angola in 1857, an infection transmitted by soft ticks (endemic tick-borne relapsing fever). Obermeier saw spirochetes, now recognized as *B. recurrentis*, in the blood of febrile patients in Berlin in 1866 [9]. In Eurasia, RF transmitted by ticks was first described by Dschunkowsky [10]; later, the microorganism was named *Borrelia persica*. Dramatic epidemics of louse-borne relapsing fever (LBRF) responsible for several millions of cases and a high fatality rate occurred throughout Africa after World Wars I and II, when French and British colonial soldiers infected in Europe or North Africa returned to their countries [11].

In addition to febrile episodes, symptoms include tachycardia, headache, conjunctivitis, hepatomegaly, splenomegaly, discoloration of the urine, asthenia, vomiting, myalgia, and arthralgia. Diagnosis is based on patient history, physical examination, and May–Grunwald–Giemsa staining of blood smears for microscopic confirmation of spirochetes. Morphology alone does not allow distinguishing spirochetes, which can be identified by molecular methods such as PCR and sequencing [12]. The mortality of tick-borne relapsing fever is generally low and can be linked to the Jarisch–Herxheimer reaction, which occurs in less than half of cases, but can also manifest itself with very serious symptoms [13].

RF borreliosis is well known and common in the African continent, but unfortunately, many African laboratories are not able to perform biomolecular tests. Therefore, the exact species distribution and potential animal reservoirs are often unknown. Within central, Southern, and Eastern Africa, *Borrelia duttonii* has mainly been described, while in northernmost Africa, *B. crocidurae* and *B. hispanica* can also be found as human pathogens [14]. According to vectors, *Borreliae* of the relapsing fever group can be divided into four subgroups, as described in Table 1.

## 2. Endemic Relapsing Fever Borreliosis (STBRF)

Soft-tick-borne relapsing fever (SBRF) was first recognized in Eastern and central-southern Africa in 1904 [15], in North Africa in 1928 [16], and in West Africa in 1932 [17].

Vectors of the STBRF are argasid ticks of the genus *Ornithodorus* sp., which lack the dorsal shield.

Hosts and reservoirs for these *Borreliae* are animals such as porcupines, foxes, rodents, and monkeys, while man is an occasional host [18], except for *Borrelia duttonii* in Africa, for which no animal has been identified as reservoir. It is also possible that the *Ornithodoros moubata* tick itself acts as both vector and reservoir [19].

STBRF spirochetes adapt and colonize the salivary glands of *Ornithodoros* sp. in the long term and are maintained trans-stadially and transovarially. Transmission of *Borrelia* after tick bite occurs within a few seconds. A notable feature of their vector biology is the specificity of a given species of STBRF for a specific species of *Ornithodoros*. This peculiarity allows one to know the distribution of the *Borrelia* spp. based on its vector.

STBRFs are well known and common in the African continent [20]. Typical habitats for soft ticks are caves and burrows of rodents and small mammals in forested mountains at altitudes over 900 m. In Northern countries, *O. tholozani* also lives in houses and stables where the floor is soil. The infection is transmitted to humans and animals through the bite of the soft tick, and the spirochete is inoculated into the new organism at the end of the meal through the regurgitation of saliva. A blood meal lasts from 15 to 90 min and is usually carried out at night.

While several tick saliva proteins have been characterized in hard ticks and have been shown to be essential in the transmission of pathogens, very few have been identified in *Ornithodoros* sp. Similarly, the process of transmission and persistence of RF bacteria in the vertebrate host is not clear, although antigenic variations and erythrocyte rosetting have been described as potential virulence factors. The first study made on *Ornithodoros*’ saliva showed its anti-hemostatic activity [21]. Subsequently, with advances in proteomics and transcriptomic techniques, some further investigations on the saliva of *O. moubata* and *O. erraticus* were published [22]. The saliva of *Ornithodoros* sp. supports the feeding process by providing a cocktail of anti-hemostatic, anti-inflammatory, and immunomodulatory molecules [23]. The salivary transcriptome of the *Ornithodoros parkeri* soft tick includes genes of the lipocalin family, as well as certain genes with Kunitz domains indicative of serine protease inhibitors. *Ornithodoros sonrai*, *O. erraticus*, or *O. normandi* ticks have been collected in burrows in Morocco, Algeria, Tunisia, Mauritania, Senegal, Gambia, Mali, and Spain [24]. In all countries where *Ornithodoros* ticks have been found, the distribution is generally contiguous, and the burrows are located near the floodplain of the Niger River [24].

### 2.1. Epidemiology

The STBRF *Borreliae* are conventionally divided geographically into the “Old World” strains, including *Borrelia crocidurae*, *Borrelia duttonii*, and *Borrelia hispanica*; and the “New World” strains, with *Borrelia hermsii*, *Borrelia parkeri*, and *Borrelia turicatae*.

#### 2.1.1. Old World Strains

Details on Old World strains are reported in Table 2. The four main species responsible for STBRF in Europe are: *B. hispanica*, *B. persica*, *B. caucasica*, and *B. crocidurae* [25]. In Eurasia, relapsing fever is sporadic, mainly affecting those who involuntarily enter tick-infested caves, ruins, or animal shelters. Most infections are associated with *Borrelia persica* transmitted by the bite of the *Ornithodoros tholozani* tick [26]. About 30–60% of *O. tholozani* ticks from Israel were reported to carry this spirochaete. As for other Ornithodoros ticks, this species has great longevity, surviving between 5 and 10 years of fasting [27]. In Eurasia, other species that cause recurrent fever coexist with *Borrelia caucasica* and *Borrelia latyschewii*. The taxonomic position of these species and other relapsing fever spirochetes is not known. In Western European countries such as Spain and Portugal, RF is often caused by *Borrelia hispanica* [28]. Its vector is *Ornithodorus erraticus*, a soft tick that is usually found in old places with pigs’ herds. In Portugal, the first human case of tick-borne relapsing fever (TBRF) was reported in 1942, but up to the early 1960s, the disease had rarely been described [29,30].

The infection is sporadic, usually following an opportunistic infection in individuals exposed to ticks. Furthermore, the infection has been seen as an imported disease in those traveling from endemic regions [31]. For this reason, in patients with recurrent episodes of fever, it is important to consider this diagnosis if patients have recently traveled to Africa, America, or the Middle East [32]. New species have also been described, but their clinical relevance remains to be clarified [33].

**Table 2:** Old World soft-tick-borne relapsing fever *Borreliae* with geographical areas and vectors.

*Borrelia*	*Ornithodoros* Tick	Geographical Areas	Disease (Reservoirs)	References
*B. armenica*	*O. verrucosus*	Ukraine	Mouse infection (mouse, guinea pigs)	[34]
*B. babylonensis*	*O. verrucosus*	Russia, Ukraine	Mouse infection	[35]
*B. baltazardii*	*O. tholozani*	Iran	STBRF	[36,37]
*B. caucasica*	*O. verrucosus*	Ukraine, Caucasus, southeast Europe	STBRF	[34,38]
*B. crocidurae*	*O. marocanus*	Western and Northern Africa	STBRF mild symptoms	[39]
*B duttonii*	*O. moubata*	Central, Eastern, and Southern Africa	STBRF neurological signs, neonatal infection	[40,41]
*B. graingeri*	*O.* *graingeri*	Kenya	Flu-like syndrome	[42]
*B. harveyi*	unknown	Kenya, East Africa	Bacteremia of monkeys (*Cercopithecus aethiops*)	[43]
*B. hispanica*	*O. erraticus*	Iberian Peninsula and Northern Africa	STBRF Ocular and neurological symptoms	[32]
*B. kalaharica*	*O. savignyi*	Kalahari desert (Botswana and Namibia)	STBRF	[44]
*B. latyschewii*	*O. tartakovsky*	Iran, Middle East	STBRF	[45]
*B. mazzottii*	*O. talaje*	Mexico, Central America, and western USA	STBRF	[46]
*B. merionesi*	*O. costalis* and *O. merionesi*	Morocco and Atlantic coastal areas of the Sahara desert	Non-infectious to humans (rodent, monkeys)	[24,47]
*B. microtti*	*O. erraticus*	Iran, Afghanistan, Eastern Africa	STBRF	[48]
*B. persica*	*O. tholozani*	Central Asia, Middle East, Egypt, and India	STBRF, infectious for dogs and cats	[49,50]
*B. tillae*	*O. zumpti*	Southern Africa	Rodents	[51]

STBRF—soft-tick-borne relapsing fever.

Cases of STBRF after arthropod bite have been reported in the Kalahari desert. The causative agent is a new RF *Borrelia*, referred to as “Candidatus *Borrelia kalaharica*”, which shares more homologies with New World recurrent fever *Borreliae*, such as *B. parkeri* and *B. hermsii*, than with Old World species, such as *B. duttonii* or *B. crocidurae* [52].

*Borrelia baltazardii* was isolated from *O. tholozani* in a region of Iran where *B. persica* had already been known, from which it differs regarding its pathogenicity in animals. The name of *Borrelia baltazardii* has been proposed for this new *Borrelia* sp. nov. [36,37]. *B. merionesi*, first isolated in *Meriones shawi* in 1937, was recognized as a new species by Blanc and Maurice due to differences in pathogenicity for laboratory animals and humans in comparison to *B. hispanica*, *B. crocidurae*, and *B. duttoni* [47].

##### Geographical Distribution

In Central Asia and Middle Eastern countries, STBRF is mainly caused by *Borrelia persica*, and to a lesser extent by *B. microtti*, *B. latyschewii*, *B. baltazardi*, and *B. caucasica* [33]. In Iran, several species have been associated with human STBRF, including *B. microtti*, *B. persica*, and another genotype close to *B. duttonii* and *B. recurrentis* [34].

Tick-borne relapsing fever was first recognized in East Africa in 1904 [35], in North Africa in 1928 [36], and in West Africa in 1932 [26]. Studies made between 1905 and 1960 have progressively established the classic picture of STBRF in Africa, with three different vector/pathogen complexes involving soft ticks (Argasidae) of the genus *Ornithodoros* sp. In the eastern Savannah areas (Tanzania) and Southern Africa (Eritrea, South Africa), STBRF is caused by *Borrelia duttonii*, which has *O. moubata* and *O. porcinus* as vectors [37]. In nature, these two ticks live in large burrows of ants, warthogs, and porcupines, and they have subsequently adapted to human dwellings and pet shelters, where they live in the crevices of walls and floors. There are no known mammalian reservoirs, and *O. moubata* enables *Borrelia* spp. to survive for very long periods through vertical transmission. It seems that *O. moubata* can act as both vector and reservoir of *B. duttonii* [53]. The annual incidence of the disease is very high in children younger than 5 years, especially in Tanzania. *B. duttonii* causes several clinical manifestations, often with neurological involvement [38]. In North Africa, from Morocco to Algeria and Tunisia, STBRF is classically caused by *Borrelia hispanica*, with *O. erraticus* as vector and small mammals as reservoir hosts [39]. *O. erraticus* is found in both large and small burrows and under stones, and has adapted to pet shelters. Most human infections occur during summertime in people sleeping in fields or farm buildings [40]. In West Africa, in Senegal and other countries, STBRF is caused by *Borrelia crocidurae*, with *O. sonrai* as vector and rodents and insectivores as reservoir hosts [25]. *B. crocidurae* infection is indeed the most common bacterial infection in this region [41]. *O. sonrai* inhabits rodent burrows and, as with other *Ornithodoros* sp., feeds quickly. Blood meals last only a few minutes. People are usually infected when sleeping, with burrows opening in their bedrooms [42]. In Africa there is a high incidence of STBRF, which contrasts with the low number of reports. This infection is maybe not often recognized, or it may be confused with malaria fever, which is an important differential diagnosis [25].

#### 2.1.2. New World Strains

Details on New World strains are reported in Table 3. In the United States, the following specificities are observed: *Borrelia hermsii* with *Ornithodoros hermsi*, *Borrelia parkeri* with *Ornithodoros parkeri*, and *Borrelia turicatae* with *Ornithodoros turicata*. *B. hermsii*, *B. parkeri*, and *B. turicatae* are predominant and have been identified and studied in the western part of the US [43].

*Borrelia braziliensis* was first reported by Davis in 1952 in Brazil. It is transmitted by an *Argasid* sp., which lives underground in the basements of houses and farms, where the floor is the natural ground and hygienic conditions are poor. In addition of transmitting STBRF, it introduces a poison by its bite, which can cause a toxic reaction associated with severe symptoms [54].

*B. johnsonii*, a new tick-borne relapsing fever *Borrelia*, was found in the bat tick *Carios kelleyi.* It is more closely related to *B. turicatae* and *B. parkeri* than to *B. hermsii*, but is clearly distinct from them [55]. The human health implications of this new relapsing fever group spirochaete are not yet known [55]; however, recently *B. johnsonii* was detected in a patient reporting tick-borne illness symptoms [56]. The name *B. johnsonii* was given in honor of Dr. Russell C. Johnson [57].

**Table 3:** New World soft-tick-borne relapsing fever *Borreliae* with geographical areas and vectors.

*Borrelia*	*Ornithodoros* Tick	Geographical Areas	Hosts and Disease	Ref.
*B. braziliensis*	*O. braziliensis*	Plateau of southern Brazil	STBRF (dogs, armadillos)	[54]
*B. coriaceae*	*O.* *coriaceus*	Western North America, northwest California	Deer bacteremia, dogs, bovine epizootic abortion	[58]
*B. dugesii*	*O. dugesii*	Mexico	(Rodents, *Neotoma micropus*)	[59]
*B. hermsii*	*O. hermsi*	Western North, White Mountains (Arizona, US), British Columbia (Canada)	STBRF (rodents, squirrels)	[60]
*B. mazzottii*	*O. talaje*	Mexico, Central America, and western US	STBRF	[46]
*B. parkeri*	*O. parkeri*	Western US	STBRF	[61,62]
*B. queenslandica*	*O. gurneyi*	Australia	Bacteremia with relapse (Rodents, *Rattus villosissimus*, Kangaroo)	[63]
*B turicatae*	*O. turicata*	British Columbia (Canada), southwestern and south-central US and Mexico	STBRF	[64,65]
*B. venezualensis*	*O. rudis*	Central America and northern South America, Venezuela, Brazil, Colombia, Panama	STBRF	[66]
*B. johnsonii*	*Carios kelleyi*	Canada, US, China, Mexico, Cuba, Costa Rica, Chile	Bats, tick-borne illness to be defined	[56,67,68]

##### Geographical Distribution

STBRF is endemic in the western United States, predominately in mountain regions, in 12 US states, namely Arizona, California, Colorado, Idaho, Montana, Nevada, New Mexico, North Dakota, Oregon, Texas, Utah, and Washington. Most RF cases in the US are caused by *Borrelia hermsii* and transmitted by *Ornithodoros hermsi* soft ticks, which typically live in nests of rodents such as ground squirrels, tree squirrels, and chipmunks in coniferous forests at altitudes between a 500 and 2500 m. Soft ticks can acquire RF *Borrelia* by feeding on infected rodents, the reservoir hosts. Once infected, soft ticks are infectious for life [44].

In California, sciurid rodents, including chipmunks (*Tamias* spp.) were found positive for serum antibodies to *Borrelia hermsii* [69]. *Borrelia turicatae* is the primary known causative species of STBRF at low-altitude, arid regions, in the southern US and Texas, inducing neurologic symptoms. *O. turicata* ticks are considered an arthropod reservoir of *B. turicatae* because they have a 10-year life span and might endure years of starvation while retaining the ability to transmit *B. turicatae* [46]. *Borrelia parkeri* is present in the western US, Colorado, and California, and is transmitted to humans by *Ornithodoros parkeri* ticks [47].

The first case of TBRF in Mexico was reported by Brumpt as an infestation from *B. turicatae*, which has as vector *O. turicata* [70]. Since RF is considered a forgotten and neglected tropical disease, and given the small number of cases reported by the Mexican Ministry of Health, it is conceivable that many patients who have a fever of unknown origin are suffering from RF [70]. In Mexico, the following specificities of *Borrelia* tick association are observed: *Ornithodoros turicata* with *B. turicatae*, *Ornithodoros duguesi* with *B. duguesii*, and *Ornithodoros talaje* with *B. mazzottii* [71]. In Brazil, Venezuela, Colombia, and Panama STBRF is caused by *Borrelia venezualensis*, which is transmitted by *Ornithodoros rudis* [66]. In central Chile, *Borrelia johnsonii* has been identified in *Ornithodoros atacamensis* ticks, infesting small mammals in a national reserve in the Andes Mountains [72]. Imported cases of STBRF have also been described in the literature. Infections have been acquired in West Africa (Senegal, Mali, Mauretania, Gambia), East Africa (Ethiopia), North Africa (Morocco), or Central Asia (Uzbekistan, Tajikistan). In most cases, the causative species was *Borrelia crocidurae* (acquired mainly in Senegal, Gambia, Mauretania, Mali) [73]. Cases of imported *Borrelia hispanica* (acquired in Morocco) [31] and *Borrelia persica* (acquired in Uzbekistan, Tajikistan) have also been described [49].

*Carios kelleyi*, the tick bat which is the vector of *B. johnsonii*, requires prolonged feeding. Therefore, bats could cause significant spreading of this tick and its associated spirochaete over a large geographic area. *Carios kelleyi* ticks have been documented in the US, Canada, China, Mexico, and Costa Rica [64,65,71]. In addition to bats, these ticks can also feed on humans and host *Rickettsia* spp. [67], with possible implications for tick-transmitted human diseases.

### 2.2. Microbiology

RF borrelia are motile, chemo-organotrophic, microaerophilic and host-associated bacteria [74]. The spiral body tends to be shorter than in the Lyme group *Borreliae*, and it is lacking cytoplasmic tubules. Several endoflagella (15–20) wind around the protoplasmic cylinder and overlap in the middle. Sections of cells reveal a triple-layered outer membrane. Actually, most RF *Borreliae* have been adapted to BSKII medium cultivation; however, not-yet-cultivable Spirochaetes can be maintained through serial passages in vivo. Notably, 10-week-old Swiss mice can be inoculated intraperitoneally with 0.4 mL infected blood preserved in liquid nitrogen [75]. The maximal yield of spirochaetes is around 10^6^–10^7^ spirochete/mL of blood, and it is usually achieved 3 days after inoculation of infected blood. The in vivo inoculation represents an important step for the isolation of RF *Borreliae* from hosts. Strains cultivated in vitro don’t lose their infectivity in mice.

STBRF spirochetes adapt and colonize in the salivary glands of *Ornithodoros* sp. and are maintained trans-stadially and transovarially. This means that the transmission of *Borrelia* in mammals after a bite occurs in a few seconds, developing high spirochaetemia. These *Borreliae* spp. usually grow at temperatures between 33 and 35 °C, corresponding to the mammalian host temperature, but can also multiply at 22 °C (tick temperature) as demonstrated in vitro for *B. turicatae* [76]. During a blood meal, STBRF spirochetes enter the middle intestine, and in the following weeks they, in part, migrate and colonize the salivary glands, which is important for rapid transmission [77]. Spirochetes populating salivary gland are therefore predisposed to establish early mammalian infection [78].

The infectious cycle of argasid-transmitted RF spirochetes requires adaptation to three environments, namely the midgut and salivary glands of the tick and the vertebrate host.

Pathogens regulate genes involved in antigenic variation to facilitate escape from the host antibody response. The dynamics between host antibody response and antigenic variation can continue for several months, providing multiple opportunities for the acquisition of spirochetes from uninfected ticks [79].

#### 2.2.1. Phylogenetic Analysis

**Figure 1:**
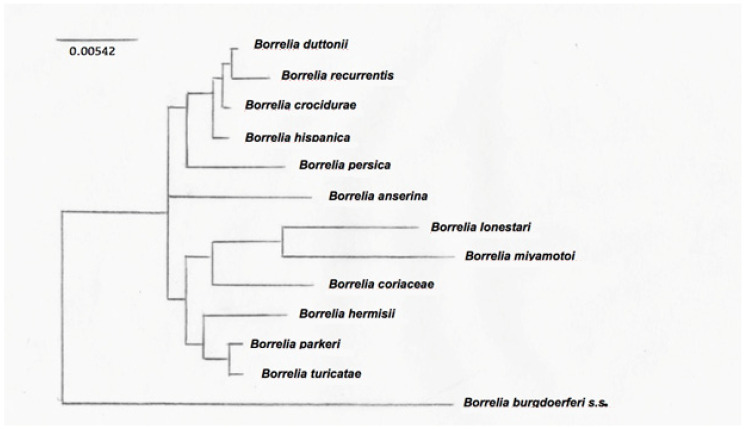
Phylogenetic tree based on the 16SrRNA sequences of the main RF *Borrelia* species. The 50% majority rule consensus tree based on 12 trees was obtained by using a maximum parsimony method [75].

The typing of RF-group *Borrelia* is challenging because of their segmented genomes, with essential genes on large linear plasmids [80]. This is further complicated by the antigenic variation. Intergenic spacer heterogeneity (IGS) between 16S–23S genes is resolutive to discriminate between Lyme group and some relapsing fever *Borreliae* [80], but not for clustering RF *Borreliae*. The phylogenetic tree derived from nucleotide sequences of the *flaB* gene clearly separates New World from Old World RF *Borreliae* [81]. Nevertheless, for a more precise classification of the different RF *Borreliae*, non-coding intragenic spacer regions are more useful for their variability, because they were not under selective pressure and thus probably reflect changes accumulated over time, without functional constraints [82]. Differences obtained from gene-sequence-based comparisons more likely reflect the accumulation of adaptive changes due to different vectors for transmission, therefore allowing the subgrouping of RF *Borreliae* in common species groups [82]. Phylogenetic trees have been obtained among a limited number of species, being the registered data of sequences not completely available. An example of a phylogenetic tree is shown in Figure 1, where three clusters of RF *Borrelia* have been identified on the basis of the analysis of 13 species representative of the main RF *Borreliae*.

#### 2.2.2. Genome

RF *Borrelia* species have segmented genomes and maintain essential genes on large linear plasmids [80]. The genome size of RF *Borreliae* (1–1.5 Mb) is smaller than the other pathogenic bacteria with a more versatile lifestyle (e.g., *P. aeruginosa*, 6.3 Mb). This 160 kb linear mega-plasmid has fairly conserved syntony between *B. duttonii* (lp165), *B. hermsii* (lp174), and *B. turicatae* (lp150), which are not found in the *Borreliae* Lyme group [83].

Genes encoding enzymes for the synthesis of most amino acids, fatty acids, enzyme cofactors, and nucleotides are absent in RF *Borreliae* genomes, as shown in Lyme group *Borreliae* [84]. RF *Borrelia* genomes have a limited repertoire that reflects very well their adapted lifestyle, including only a few genes associated with virulence.

The Argasidae and Ixodidae cuticle, which contains chitin derived from the polymerization of N-Acetyl Glucosamine (NAG), could be an important source of nutrients for *Borreliae* during the arthropod-associated phase [85]. Evolution by genome reduction is well correlated with the degenerate lifestyle of several well-adapted pathogens [86], which probably include RF *Borreliae*, which have a narrow niche in the vertebrate vector and host. *B. turicatae*’s mega-plasmid undergoes a shift in its transcriptional profile between in vitro tick-like growth conditions and infected murine blood, identifying a cluster of encoding putative surface lipoproteins likely involved in vector colonization and host–vector interactions [76].

#### 2.2.3. Species Identification of the STBRF

*Borrelia* species were initially distinguished on the basis of geography and vectors. This classification was based on a co-specialization hypothesis, which postulated that only one species of relapsing fever *Borrelia* could be found in a particular host and vector in a given geographic area. However, recent evidence showed the coexistence of *B. duttonii* and *B. crocidurae* in Togo and of *B. crocidurae* and *B. hispanica* in North Africa; therefore, previous geographic distribution studies were not exhaustive [47].

Molecular typing of species can be carried out by restriction fragment length polymorphism through the amplification (PCR) of *16S-rRNA* genes and/or species-specific PCR of the glycerophosphodiester phosphodiesterase (*GlpQ*) gene [87], which seems to be more variable than 16S-rRNA [88].

At least 10 different species of *Borrelia* with STBRF have been documented in Africa, including four species that infect humans, namely *B. hispanica, B. crocidurae, B. duttonii*, and *B. recurrentis.* Other species have been found in non-human hosts. The loci used for the initial species determination were 16S rRNA and flaB. Although they are highly conserved (especially 16S) and may have low discriminatory power in RF species, they are very useful for a first approximation of species assignment, because they have been used for many STBRF species and strains, therefore many sequences are available [89].

#### 2.2.4. Antigens

The 40 KDa *Borrelia* A repeat protein (BrpA) is characterized by the repetition of a particular amino acid motif. Deletion of the *BrpA* gene neither avoided infection of inoculated mice, nor inhibited further colonization of the *O. turicata* salivary glands and subsequent transmission [90].

Many virulence factors described for the *Borreliae* Lyme group are also present in RF *Borreliae*. The initial phase of the passage of the RF *Borrelia* from the vector to the mammal and to man (i.e., from the initial deposition of the spirochaetes on the skin to the subsequent colonization in various tissues) exposes the spirochaetes to host defense mechanisms, which *Borrelia* tries to neutralize by developing adaptation mechanisms. RF and Lyme group *Borreliae* share many characteristics [91], such as the general organization of the genome with a well-preserved tuning of the chromosome. Notwithstanding, there is an evident difference in linear and circular plasmids. The RF group has a more variable pathological spectrum, including RF transmitted by ticks and lice as well as the different and specific types of avian and bovine *Borreliae*. There is also a difference in the disease that separates these two types of spirochaetes: TBRF is characterized by high spirochetaemia, while Lyme borreliosis is characterized by tissue tropism (except for *Borrelia mayonii*), in particular the skin, joints, and nervous system [92]. They also differ in vectors, usually *Ixodes* sp. in Lyme, and several vector types in RF (soft ticks, hard ticks, louses) with transovarial transmission of *Borrelia*, especially in ticks. The tick bite is often painless, as the tick’s saliva contains pain-relieving substances as well as anticoagulant factors and anti-inflammatory substances. Additionally, tick saliva inhibits polymorphonuclear leukocytes (PMN) activity, which further reduces killing of the spirochaete [93]. Hence, RF *Borrelia* must penetrate both the extracellular matrix (ECM) and the endothelial lining of blood vessels to maintain and spread the infection to adjacent tissues. Furthermore, the use of host proteases supports the diffusion of the RF spirochaete [94]. The activation of matrix metalloproteinases (MMPs) also follows the binding and activation of plasminogen on the surface of the spirochaetes [95].

In the early stages, STBRF is a blood-borne infection with high spirochaetemia. Several species, such as *B. duttonii*, *B. crocidurae*, and *B. hispanica*, often interact with erythrocytes, causing red blood cells to clump together. This interaction allows the spirochaete to access nutrients, nestling and hiding them from the immune response [96]. RF group *Borreliae* have specific genes that allow using purines from serum as metabolites for the synthesis of different macromolecules [97]. These genes are lacking in the *Borrelia* Lyme group, which does not reach high spirochaetemia (except for *Borrelia mayonii*). RF *Borrelia* interactions with circulating cells could be a virulence strategy to increase and lengthen the time this pathogen can be retained in the host. The most important virulence factor of RF *Borrelia* is the mechanism of antigenic variation of the membrane proteins, located on the surface (variable major protein—Vmp), which allows escaping the antibody response, with a mechanism similar to the VlsE in the *Borrelia* Lyme group [98]. After the feverish peak there is an antibody response, and subsequently, *Borreliae* with this modified antigen are generated, allowing for a further spirochaetemia with a new feverish peak [99]. This process of antigenic variation can be repeated several times: each relapse corresponds to the increase of a new immunogenic variant of RF *Borreliae* that hosts a modified “main variable protein” (Vmp) on its surface. There are two different families of Vmps: large variable proteins (Vlps) of about 40 KDa and small variable proteins (Vsps) of about 20 KDa. Both Vlps and Vsps families are encoded on linear plasmids [100].

The progression of RF involves tissue invasion and colonization associated with further escape from the immune defense [18]. Several mechanisms of gene conversion, DNA rearrangements, mutations, and change in the transcription locus seem to be involved in the replacement of the active *vsp* or *vlp* gene [101]. The repertoire of genes encoding Vmp differs highly among genomes of *B. recurrentis, B. hermsii* [102], and *B. duttonii* [103].

The mechanism of antigenic variation is likely to be a common feature to all the different RF *Borreliae*. Up to now, these multiphase changes have been demonstrated for *B. hermsii* and *B. turicatae* [104]. Vsps and Vlps are phylogenetically related to the surface proteins of the *Borrelia* Lyme group OspC and VlsE, respectively [105].

#### 2.2.5. Clinical Aspects

Incubation lasts approximately 1 week; between the tick bite and the first STBRF symptoms, a length of about 3–10 days is observed [106]. An initial febrile episode with high fever with temperature ranging from 38.7 to 40/41 °C is followed by a series of relapses (3–5 in LBRF and 9–13 in STBRF) interspersed with remissions of a few days [107]. Clinical manifestations vary in severity among patients and in some cases, if left untreated, the disease can be fatal. It is usually more severe in young children [8]. The first period of fever is usually the longest and lasts about 4–7 days. The following relapses correspond to spirochaetemia peaks spaced out by a few days of remission [107]. In addition to high fever, typical symptoms include malaise, headache, neurological symptoms, myoarticular pains, nausea, vomiting, and diarrhea. Patients may have petechial-type skin rashes, different from erythema migrans, which is characteristic of Lyme group *Borreliae* [91,92]. During the spirochaete peak, more or less severe bleeding can be observed, and in some cases hemorrhages of the retina and brain. Internal organs, such as the liver, with jaundice, and the spleen can also be affected, causing enlargement and rupture. Respiratory symptoms with cough and myocarditis may also be observed [60].

Neurological symptoms are common during RF, but are more severe for *B. duttonii* and *B. turicatae* infection due to their greater neurotropism [108]. The most common symptoms are indeed meningitis and facial palsy. Ocular complications have also been reported in some cases. Anemia and thrombocytopenia are also observed, and in untreated patients a progressive worsening of general conditions can be observed, with asthenia and weight loss [108]. RF spirochaetes are susceptible to broad-spectrum antibiotics. However, upon treatment, 54% of patients develop Jarisch–Herxheimer reaction [13].

According to international organizations reports, around 10–15% of neonatal deaths worldwide are caused by serious infections [109], including tick-borne RF in endemic regions. Spirochaetes can cross the placental barrier, causing congenital infections [110]. The consequences and complications of these cases of borreliosis in pregnancy can be mild, with a slight decrease in birth weight and preterm delivery, but also severe, resulting in miscarriage or death of the newborn and pregnant woman [111]. In Rwanda, the death rate among pregnant women suffering from the disease is 16% [112]. In STBRF caused by *B. duttonii*, intrauterine growth retardation, placental damage and inflammation, impaired fetal circulation, and maternal anemia have been described.

In the United States, TBRF in dogs is caused by *Borrelia turicatae* and *Borrelia hermsii*, which are transmitted by *Ornithodoros* spp. ticks. The distinctive diagnostic feature of this infection is the visualization of several spirochaetes in standard blood smear examination. Although the course of the spirochaetemia has not been fully documented in dogs, it likely evolves with episodes of intermittent spirochaetemia and fever as in human infection [113].

In addition to infection in dogs, cases of TBRF have been reported in horses, in which it can cause abortion [114].

## 3. Hard-Tick-Borne Relapsing Fever (HTBRF)

HTBRF *Borreliae* are transmitted by several genera of hard ticks, notably *Ixodes* sp., *Amblyomma* sp., *Dermacentor* sp., and *Rhipicephalus* sp. Human infection has surely been documented for *Borrelia miyamotoi*, which was not the first *Borrelia* of the RF group transmitted by a hard tick and not by a soft tick (*Ornithodoros* sp.) to be described. More than a century ago, Arnold Theiler reported that *Rhipicephalus* sp. transmitted a spirochaete (*Borrelia theileri*) to cattle [115]. Recently, *B. lonestari* has been discovered in *Amblyomma americanum* [116]. Nucleotide sequences of *B. miyamotoi* isolates in Japan and North America confirmed that *B. miyamotoi* and the other aforementioned species are phylogenetically linked to HTBRF [117,118]. HTBRF *Borreliae* strains correlate with those of vector species rather than geographical distance [119]. Therefore, the genetic diversification of HTBRF *Borreliae* can be due to the speciation of vector ticks, and this relationship might be required for efficient transmission of HTBRF *Borreliae* within its vector [119]. Recently, the hypothesis of a common ancestor of *Borreliae* transmitted by hard-body ticks was formulated. Accordingly, STBRF *Borreliae* switched to use soft-bodied ticks as a vector, which was followed by the emergence of *Borrelia recurrentis*, the louse-borne RF *Borrelia* [119]. HTBRF *Borreliae* are summarized in Table 4 with their vectors.

*Borrelia miyamotoi* is a spirochaete of the RF group, transmitted by the same hard ticks (*Ixodes* sp.) of the *Borreliae* Lyme group [128]. It was identified in Japan in 1994 from *Ixodes persulcatus* ticks collected from the small Japanese country mouse *Apodemus argenteus* [129]. The name of this *Borrelia* was given in honor of Kenji Miyamoto, who first reported this spirochaete in Hokkaido, Japan. In 2000 in the northeastern US, a *Borrelia* closely related to *B. miyamotoi* was identified in *Ixodes scapularis* ticks. As early as 1985, in the United States spirochaetes had been observed in ticks, probably related to *B. miyamotoi* but mistakenly identified as *B. burgdorferi* due to the cross-reactivity of the antibodies used in serological tests. These spirochaetes were present in the ovarian tissue of adult ticks, eggs, and larvae of *Ixodes scapularis* and *Ixodes pacificus* and led to the false conclusion that *B. burgdorferi* could be transmitted transovarially by ticks [130]. Recent evidence has confirmed the transovarial (vertical) transmission of *B. miyamotoi* (similarly to the other RFG *Borreliae*), but not of *B. burgdorferi* in *I. scapularis* [131]. *B. miyamotoi* was later identified in other *Ixodes* sp. ticks, which are vectors of Lyme disease in Asia, North America, and Europe [132]. The discovery of *B. miyamotoi* expands the geographic range of RF group *Borreliae*, which are classically transmitted by soft. ticks (Argasidae) and lice, have different ecological niches, and are only occasionally found in the same geographic areas of the vectors of Lyme disease [133].

### 3.1. Epidemiology

Although the novelty and wide geographic distribution of *B. miyamotoi* have been recognized for several years, this spirochaete has received relatively little attention until human cases of relapsing febrile-like illness from *B. miyamotoi* infection were reported in 2011 in Russia, and later in the United States and Europe [134]. This spirochaete has a global distribution and circulates together (co-infection) with the Lyme borreliosis agent, *B. burgdorferi* s.l., which shares the same ticks (*Ixodes* spp.) as vectors [123].

*Borrelia miyamotoi* infection in ticks and hosts has been reported in Japan and Russia (*Ixodes persulcatus*), in the eastern and upper Midwest United States (*Ixodes scapularis*), in the western US *(Ixodes pacificus* in California), and in Europe (*Ixodes ricinus*). A few cases were reported in humans in China, but due to the increment of patients, this bacterium is considered an emerging pathogen [135]. In Italy the first detection of *B. miyamotoi* in *Ixodes ricinus* ticks was dated 2018 and referred to ticks collected in two independent studies in 2016 in the north-eastern and north-western Alps [136]. These reports highlight the importance of *B. miyamotoi* for public health [137]. Antigenic cross-reactivity in immunoassays between *Borrelia* species in North America can complicate the diagnosis of both Lyme disease and relapsing fever [138].

### 3.2. Hosts and Reservoirs

In Table 4, the main information about HTBRF *Borreliae* is summarized. In 1996, phylogenetic analysis of *Borrelia* DNA sequences from *Amblyomma americanum* led to the identification of *Borrelia lonestari* sp. nov., related to the RF group and not to the Lyme group [116]. Sequences of *B. lonestari*, found mainly in the southeastern US, have also been detected in ticks in the northern US, due to the fact that *Amblyomma americanum* is a parasite of migratory water birds and non-migratory wild turkeys (*Meleagris gallopavo silvestris*) [139]. The host of *Borrelia lonestari* is likely the white-tailed deer (*Odocoileus virginianus*), which is a reservoir host for several pathogens associated with *A. americanum* [120]. This deer can develop bacteremia after *B. lonestari* inoculation [121]; however, other studies have not confirmed this hypothesis [122].

Little is known about *B. miyamotoi* reservoirs, but it can be hypothesized that common characteristics are shared with bacteria belonging to the *B. burgdorferi* sl complex: small rodents such as *Apodemus argenteus* in Japan [129], *Peromyscus leucopus* in North America [140], *Apodemus flavicollis* in Europe [141], *Myodes glareolus* in Northern Europe [142], and also passerine birds [143] are responsible for the large distribution of this *Borrelia*. Sequence analysis of *B. miyamotoi* from North America [117] and Japan a confirmed *B. miyamotoi* as an RF *Borrelia* [118]. *B. miyamotoi* can be clustered in Japanese/Russian genotypes [144], which differ from European and North American ones [145]. It has been shown that in Estonia, the Asian genotype of *B. miyamotoi* could be associated with both *I. ricinus* and *I. persulcatus* ticks [146]. *B. miyamotoi* is infectious and pathogenic for humans and evades human antibodies through the mechanism of antigenic variation, which is a common feature to all *Borreliae* (Lyme and RF group) [147].

### 3.3. Clinical Aspects

The clinical manifestations in humans are characterized by fever, chills, nausea, headache, myalgia, skin rash, and lymphadenopathy. In immunocompromised patients, meningoencephalitis also can be observed. Unlike STBRF, a small number of patients experienced more febrile episodes. *B. miyamotoi* and *B. burgdorferi* (and other pathogens transmitted by *Ixodes* spp.) can simultaneously infect ticks, reservoirs, and humans. Spirochaetemia is also lower than in patients infected by STBRF *Borreliae*. Laboratory tests for *B. miyamotoi* infections show leukopenia, thrombocytopenia, and elevated liver enzymes. Spirochaetes can be observed in the acute phase in blood smears stained with May–Grünwald–Giemsa. Two-step serology for *B. miyamotoi* includes ELISA and Western blot. Glycerophosphodiester phosphodiesterase (GlpQ) was chosen as target because of its absence in the *Borrelia* Lyme group. GlpQ is found in other relapsing fever *Borrelia* species. However, this could represent a specificity problem in areas where other relapsing febrile spirochaetes are enzootic. In the acute phase, serum samples should be collected within 7 days of symptoms onset, while in the convalescent phase, they should be collected approximately 3 weeks after symptoms onset. Currently, blood PCR is the most specific test for *B. miyamotoi*, because of the high spirochaetemia in the acute phase. In vitro cultivation of *B. miyamotoi* was reported in modified and serum-supplemented Kelly–Pettenkorfer medium [148].

*Borrelia lonestari* was found in both *A. americanum* removed from a patient with the so called southern tick-associated rash illness (STARI), initially referred as Lyme-like illness, and in a biopsy of the rash [149], leading to the assumption that it was the causative agent of STARI, which manifests itself after *A. americanum* bites [150]. However, the association between *Borrelia lonestari* and STARI, and therefore human infection, has not yet been confirmed. STARI is nowadays clearly linked to *A. americanum* tick bites, but further evidence is needed to link *B. lonestari* to STARI [151].

## 4. Louse-Borne Relapsing Fever (LBRF)

Louse- borne RF (LBRF) is an epidemic disease linked to war, famine, refugees, poverty, and poor sanitation. In previous centuries, it caused severe epidemics around the world. More recently, certain epidemics occurred in Sudan. LBRF still persists in the mountains of Ethiopia, where it is endemic. Nowadays, it is a forgotten disease in Western countries, but refugees from Africa to Europe could recall it [3]. The epidemic form is caused by *B. recurrentis*, for which the vector is the human body louse (*Pediculus humanus humanus* or *Pediculus humanus corporis*). Human body lice transmission was demonstrated by Mackie in 1907 [152]. It occurs from human to human through lice, which ingest infected blood and transmit the spirochaetes to a new host through the skin or mucous membranes when the body of the louse itself is crushed by scratching [1].

### 4.1. Epidemiology

In Ethiopian highlands and in Somalia, there are still annual epidemics of thousands of cases coinciding with the rainy period. In Sudan, in 1999–2000, 20,000 cases with around 2000 deaths were reported [3]. In 1985, in the Andes of Peru, at altitudes above 3800 m, 60 cases were reported among the inhabitants of villages infested with lice [3]. In the second half of 2015, more than 40 cases of LBRF were imported into central Europe by migrants [153].

Since July 2015, LBRF has been diagnosed in about one hundred refugees arriving in Europe, mainly from Ethiopia, Eritrea, Somalia, and other African countries, representing the most frequently reported infection in Eritrean immigrants [3]. Two patients diagnosed in Turin, Italy, had been residing in Italy for years and shared accommodation with recently arrived immigrants, suggesting the possibility of native infection [154].

### 4.2. Transmission

The transmission of *B. recurrentis* is limited to one vector, the human body louse *Pediculus humanus corporis*. Fluid from a crushed louse, or louse feces infected with *B. recurrentis*, can be inoculated through broken skin or mucous membranes through scratching. Lice, differently from ticks, cannot infect their progeny; therefore, they do not act as reservoirs. No animal reservoir is known, therefore the persistence of infection between outbreaks can occur only through mild or asymptomatic human infections. *B. recurrentis* has adapted to lice transmission. Phylogenetics and sequencing data suggest that *B. recurrentis* evolved from *Borrelia duttonii* [103]. The louse vector transmission seems to be less robust than that of the ticks. Transovarial transmission has not been demonstrated. In addition to the human body louse, the *Pediculus humanus capitis* was supposed as a possible vector, since *B. recurrentis* and *B. theileri* were detected in African pygmies’ head lice [155].

### 4.3. Microbiology

LBRF is caused by *B. recurrentis*, a large, mobile, loosely coiled spirochaete with tapered ends, 12–22 μm long and 0.2–0.6 μm thick, with an average wavelength of 1.8 μm, and 8–10 periplasmic flagella [156]. *B. recurrentis* can be cultured in vitro in serum-supplemented Barbour–Stoenner–Kelly (BSK) medium [157] and Kelly–Pettenkofer modified medium (MKP) [158], with higher isolation rate, morphology, and motility in MKP-based cultures [159].

*B. recurrentis* has the simplest genome among *Borreliae*, composed of one linear chromosome of 1 Mb, 7 linear plasmids, and 990 protein-coding genes. Furthermore, it also has low genetic variability [158]. The *B. recurrentis* genome is similar to the *B. duttonii* one, but it differs in genome reduction, which allowed adaptation to *Pediculus humanus humanus*. The *B. recurrentis* genome does not include RecA and RadA proteins, which are responsible for DNA repair [103]. Recently, the genome of *B. recurrentis* was recovered from the skeleton of a young woman retrieved during excavations in a cemetery in Oslo. Radiocarbon dating suggested she lived among 1430–1465. The resulting European lineage of *B. recurrentis* was discrete, showing ancestral *oppA-1* gene and gene loss at sites of antigenic variation [160].

### 4.4. Clinical Aspects

The incubation period is 4–18 (average 7) days. The attack begins abruptly, with fever rising to almost 40 °C in a few days, accompanied by stiffness. Early symptoms include headaches, dizziness, nightmares, generalized aches and joint pains, anorexia, nausea, vomiting, and diarrhea. Upper abdominal pain, cough and hemorrhages of the conjunctiva, epistaxis, and jaundice develop later. A petechial or ecchymotic rash, involving particularly the trunk, is often observed. There is an important spirochaetemia, which is mainly localized around the lumen of blood vessels of various organs, causing miliary abscesses and infarcts of the spleen with possible rupture, liver failure, and involvement of the central nervous system, up to cerebral hemorrhage. Perivascular histiocytic interstitial myocarditis is responsible for arrhythmias, up to myocardial failure [161]. Lungs and bowel are studded with petechial and sometimes massive hemorrhages [162].

Untreated seizures resolve in 4–10 days (average 5), followed by 5–9 days of afebrile remission, followed by relapses of decreasing severity (up to 5), where nosebleeds but no purpuric eruptions may occur. Pregnant women are particularly susceptible to severe illness and premature birth, which is frequent.

*B. recurrentis* Vmps, which is expressed in the host, is the main factor inducing TNF (tumor necrosis factor), which is likely involved in the Jarisch–Herxheimer reaction, which can be extremely severe with the beginning of the antibiotic treatment [163].

## 5. Avian Relapsing Fever *Borreliae*

*Borreliae* can infect birds, which can also act as reservoirs (see Table 5). The first report was made by Sakharoff in 1891 [164], who described the *Spirochaeta anserina* later named *Borrelia anserina* [165]. The micro-organism was identified as a member of the genus *Borrelia* by analyzing the *16S rRNA* gene sequence, showing characteristics of the RFG *Borreliae* [166]. *B. anserina* was also isolated in BSK culture from a domestic chicken in California [167].

*Borrelia anserina* is globally distributed, and it is the infectious agent of the avian spirochetosis, a tick-borne disease of poultry [168], with important economic concerns. This *Borrelia* makes use of *Argas* sp. as vectors (Table 5). The tick *Carios capensis* infests the nests of brown pelicans, *Pelecanus occidentalis*, and other ground-nesting birds along the coast of South Carolina. These ticks harbored *Borreliae* related to “*Borrelia lonestari*” [169]. *Borreliae* (6.8%) were recently detected in *Carios (Ornithodoros) sawaii* ticks collected from seabird nests. Sequencing analysis of *16S rRNA* gene showed that they were phylogenetically strictly related to *Borrelia turicatae* [170].

*B. anserina* differs phenotypically from the other species of the RFG *Borreliae*: it has a smaller genome, made by a linear chromosome of ~900 kb, and a mega-plasmid, like other members of the genus, but in total, fewer plasmids [171,172]. In comparison to other *Borreliae*, hosts are limited to birds. The main advantage for spirochaete in infecting avian hosts is the opportunity for spreading in wide geographic regions. The impact of avian borreliosis on its host is not fully known; however, it could be associated with mortality. Acute septicemic spirochaetosis has been diagnosed in an adult male spotted owl (*Strix occidentalis caurina*) found dead in Washington, USA. Silver-stained biopsy specimens of the liver, spleen, brain, and blood vessels showed several spiral-shaped bacteria [166].

## 6. Unclassified *Borreliae*

A possible list of unclassified *Borreliae* is reported in Table 6.

*Borrelia texasensis* was isolated in Texas (TXW-1) from the *Dermacentor variabilis*, also known as the American dog tick. The TXW-1 strain has a small 38 KDa endoflagellar protein, and it lacks the *OspC* gene. TXW-1 isolate seems to represent an undescribed species in RFG *Borreliae*. These *Borreliae* are microaerophilous and can be grown in BSK-H medium at 34 °C. They are Gram-negative and Giemsa-positive. Their pathogenicity and infectiveness in animals and humans are unknown [173].

*Borreliae* in hard ticks were found in a low percentage (1%) in the Ivory Coast, by amplifying a fragment of the *flaB* gene and the *16S rRNA* gene sequence, which were related to undescribed species similar to *Borrelia duttonii*. These new species had not previously been isolated by culture, therefore the names “Candidatus *Borrelia africana*” for the TCI22 genotype and “Candidatus *Borrelia ivorensis*” for the TCI140 and TCI351 genotypes were proposed. Based on a phylogenetic tree analysis of the *flaB* gene, the sequences of *Candidatus Borrelia africana* and *Candidatus Borrelia ivorensis* are located in the genus *Borrelia* close to that identified in *Amblyomma cohaerens* in Ethiopia (GenBank JX089967), and are closer to the RFG than to the Lyme group [174]. These potential new *Borreliae* form a new clade between *Borrelia* Lyme group clades and the RF group [175].

In Oromia (Ethiopia), 3.8% of ticks, which parasitize domestic animals, tested positive for *Borrelia* DNA. The prevalence of *Borrelia* DNA was significantly higher in ticks of the genus *Amblyomma* spp. (6.7%) compared to ticks of the genus *Rhipicephalus* spp. (2.1%). Sequencing data of the *flaB* and *16S rRNA* genes of *Borrelia* spp. from *Amblyomma* ticks showed the presence of a new intermediate species between the RFG and Lyme group *Borreliae*. The pathogenicity for humans of *Borrelia* sp. found in the *Amblyomma* ticks of Ethiopia has not yet been studied, while *Borrelia* sp. detected in *Boophilus/Rhipicephalus* ticks is the causative agent of bovine borreliosis [176].

In the UK, in a bat parasitized by the *Argas vespertilionis* tick, commonly known as the short-legged bat tick, several spirochaetes were detected. The bat showed at autopsy muscle damage, liver and splenomegaly, enlarged lymph nodes, and adrenal hemorrhage. Specific PCR analysis of fragment of the genes encoding *16S rRNA*, *glpQ*, and *flaB* highlighted differences from *B. johnsonii* [177], which has been found in bats’ ticks and also linked to human disease [55,56]. It remains to be clarified whether bats are an extension of the spirochaetic reservoir of RF or represent an enzootic cycle with only negligible significance to humanity.

In the Andean valley in Chile, *Borrelia* sp. Cachapoal was found in rodent *Ornitodoros* ticks, closely related to *Ornitodoros atacamensis*. Even if the role of this *Borrelia* as etiologic agent of RF in human is unknown, its phylogenetic position is very close to *Borrelia johnsonii* [72].

## Figures and Tables

**Table 1:** *Borreliae* of relapsing fever group subgroupings according to symptoms, reservoirs, and vectors.

Sub-Groups	Main Symptoms in Humans			Reservoirs	Vectors
	Headache	Fever	Erythema Migrans		
STBRF endemic RF	Yes	Yes	No	Rodents birds, bats, insectivorous *Ornithodoros moubata*	*Ornithodoros* sp. *Carios kalleyi*
HTBRF	Yes	Yes	No	Rodents, birds, deer (*Odocoileus virginiatus*)	*Ixodes* sp., *Amblyomma* sp.
LBRF epidemic RF	Yes	Yes	No	No reservoirs	*Pediculus humanus humanus*
Avian worldwide RF	No			Birds	*Argas* sp.

RF—relapsing fever, STBRF—soft-tick-borne relapsing fever, HTBRF—hard-tick-borne relapsing fever, LBRF—louse-borne relapsing fever.

**Table 4:** Hard-tick-borne relapsing fever *Borreliae*.

*Borreliae*	Hard Ticks	Geographical Areas	Disease (Hosts)	References
*B. lonestari*	*Amblyomma americanum* (lone star tick)	USA (Missouri, California)	Southern tick-associated rash illness (STARI) (?), (deer (*Odocoileus virginianus)*, migratory birds, wild turkeys (*Meleagris gallopavo silvestris)*)	[120,121,122]
*B. miyamotoi*	*Ixodes persulcatus*, *I. ricinus*, *I. scapularis*	Asia, Europe, USA	HTBRF (rodents, birds)	[123]
*B. theileri*	*Rhipicephalus* sp., *Margaropus australis*	Africa, Australia, Brazil, northern South America	Bovine spirochaestosis (cattle, sheep, goats, horses)	[124,125,126,127]

**Table 5:** Avian relapsing fever *Borreliae*.

*Borrelia*	Soft Ticks Vectors	Geographical Areas	Reservoirs/Hosts
*B. anserina*	*Argas* sp.	Worldwide	Birds

**Table 6:** Unclassified *Borreliae*.

*Borrelia*	Vectors	Geographical Areas	Reservoirs
*B. texasensis*	*Dermacentor variabilis*	USA (Texas)	Unknown
*Candidatus Borrelia africana* *Candidatus Borrelia ivorensis*	*Amblyomma* sp. *Rhipicephalus* sp.	Ivory Coast	
Oromia’s *Borrelia*	*Amblyomma* sp., *Rhipicephalus* sp.	Ethiopia	Domestic animals
UK pipistrelle bat associated spirochaete	*Argas vespertilionis*	UK	Bats
*Borrelia* sp. Cachapoal	*Ornithodoros* sp. Cachapoal	Chile	Rodents

## Data Availability

No new data were created or analyzed in this study. Data sharing is not applicable to this article.
